# Establishment and Biological Characteristics Analysis of a Hybrid Culter Lineage from *Megalobrama amblycephala* (♀) and *Culter alburnus* (♂)

**DOI:** 10.3390/ani15243555

**Published:** 2025-12-10

**Authors:** Jinhui Huang, Yingying Yang, Jiawang Huang, Xiaoyu Huang, Jiaxuan Zhu, Yanran Xiong, Lang Qin, Hongxuan Liang, Ming Wen, Yuxiang Wang, Xu Huang, Fangzhou Hu, Shi Wang, Chang Wu, Shaojun Liu

**Affiliations:** 1 Engineering Research Center of Polyploid Fish Reproduction and Breeding of the State Education Ministry, College of Life Sciences, Hunan Normal University, Changsha 410081, China; 2 Yuelushan Laboratory, Changsha 410082, China; 3 Institute of Interdisciplinary Studies, Hunan Normal University, Changsha 410081, China

**Keywords:** distant hybridization, hybrid lineage, ITS, MSAP, differentially expressed genes

## Abstract

*Culter alburnus* (topmouth culter, TC) is extensively distributed in various rivers and lakes in China. As a widely adaptive fish species, they have significant economic value and special ecological roles. Intergeneric hybridization is a pivotal strategy for generating novel hybrid lineages and species. In a previous study, we obtained an improved bisexual hybrid culter, BTBTF_1_, derived from the hybrid lineage of *Megalobrama amblycephala* (blunt snout bream, BSB, 2n = 48, ♀) × *Culter alburnus* (2n = 48, ♂). In this study, we established an improved hybrid culter lineage by the self-crossing of BTBTF_1_ and evaluated the biological characteristics regarding cytology, morphology, and genetics. DNA content and chromosome analyses confirmed that BTBTF_1_-F_2_ was a diploid lineage (2n = 48), with morphological traits exhibiting intermediate values between parental species, except for significantly TC-biased full-length-to-body length (FL/BL) and body length-to-head length (BL/HL) ratios (*p* < 0.05). ITS sequencing analysis revealed that BTBTF_1_-F_2_ inherited ITS1 sequences from BSB and TC. The global methylation level in BTBTF_1_-F_2_ was substantially reduced compared to progenitors, characterized by elevated full and diminished hemimethylation states. Transcriptomic analysis identified 7877 differentially expressed genes (DEGs), displaying 9.05%/8.30% maternal (BSB)-dominant and 17.01%/18.95% paternal (TC)-dominant expression patterns in BTBTF_1_ and F_2_. Remarkable intergenerational similarity in phenotypic and molecular profiles, coupled with bidirectional inheritance of progenitor characteristics, confirmed BTBTF_1_-F_2_ as a genetically stable allodiploid lineage. Remarkably, methylation patterns, and DEG expression collectively demonstrated significant TC-oriented bias (*p* < 0.05). This study reports a novel stabilized allodiploid culter lineage after a comprehensive assessment at cytology, morphology, and genetic levels, and provides new insights into genetic bias in hybrid progeny.

## Simple Summary

The topmouth culter and blunt snout bream are two economically important fish species for aquaculture. Crossing different fish species is an effective way to develop improved varieties, and we previously created a fertile hybrid named BTBTF_1_ by crossing these two species. In this study, we produced a new hybrid lineage (BTBTF_1_-F_2_) through the self-mating of BTBTF_1_ and analyzed their biological characteristics. We found that BTBTF_1_-F_2_ has the same 48 chromosomes as its parents, with morphological traits that are intermediate between the two but more similar to the topmouth culter in some aspects. It inherited genes from both parents and maintained stable genetics across generations, with lower overall genetic methylation than its parents. This stable new hybrid culter lineage benefits aquaculture and helps us understand genetic inheritance in hybrid fish.

## 1. Introduction

Topmouth culter (*Culter alburnus*, TC) is a type of fierce, carnivorous Cyprinidae species [1] living in the middle and upper layers of water. It is distributed in almost every river and lake in China [2]. As is well-known, TC has high economic value and special ecological roles [3,4], which have motivated the extensive research interests of ichthyologists. Distant hybridization, defined as crosses between distinct species or higher taxa [5], facilitates the integration of divergent genomes and generates profound phenotypic and genotypic alterations in hybrid offspring [6]. The hybrid progeny obtained are typically superior to their parents for a few characteristics, including growth rate [7] and meat quality [8], known as the hybrid vigor or heterosis [9], which is beneficial for breeding and production. Distant hybridization has been extensively used to prepare novel and improved fish types [10]. When hybridization yields fertile progeny, subsequent generations may be propagated through self-fertilization, thereby enabling the inheritance of heterosis. For instance, an improved triploid crucian carp with rapid growth rate and sterility was produced by crossing male allotetraploid lineages with female red crucian carp. This hybrid demonstrated significant economic value for aquaculture and played a crucial role in protecting wild fish resources due to its inability to mate with wild fish [11].

45S rDNA is a highly conserved housekeeping gene in eukaryotes. It serves as an effective molecular marker for analyzing the evolutionary relationships and mechanisms of species [12]. Its repeat unit consists of three rDNA transcription regions (18S, 5.8S, and 28S rDNA), two internal transcribed spacers (ITS1, ITS2), and an intergenic spacer region (IGS) [13]. ITS1 and ITS2 are minimally influenced by external factors and evolve rapidly. Most variations were independent point mutations with distinct interspecific differences. Consequently, ITS sequences are valuable molecular markers for species identification [14]. Its utility is validated in international studies on hybrid fish systematics [15].

Methylation-sensitive amplification polymorphism (MSAP), exploiting differences in the sensitivity of *Msp*I and *Hpa*II restriction enzymes to cytosine methylation for genome-wide methylation detection, has been confirmed to be feasible in numerous studies in plants [16,17] and animals [18]. RNA sequencing, established based on next-generation sequencing, performs high-throughput sequencing of all transcribed RNAs in tissue cells and is widely used in aquaculture and fisheries to study the inheritance and variation in gene expression in species [19].

In previous studies, a fertile allodiploid lineage derived from blunt snout bream (BSB, ♀) × topmouth culter (TC, ♂), known as BTF_1_-F_6_ [20], was identified. During spawning season (May–July) in 2017–2019, the new type of hybrid culter (BTBTF_1_) was successfully obtained by two rounds of back-crossing of BTF_1_, and was confirmed to be bisexual and fertile [21]. In this study, we established the hybrid lineage of BTBTF_1_-F_2_ by self-crossing of BTBTF_1_, surveying the biological characteristics of morphology, DNA content, and chromosome number, and evaluating the genetic stability of the hybrid lineage at genomic, epigenetic, and transcriptomic levels using BSB and TC as controls. The results provide important insights into the heredity and variation in the process of hybrid lineage formation or speciation.

## 2. Materials and Methods

### 2.1. Ethics Statement

The guidelines established by the Administration of Affairs Concerning Animal Experimentation state that approval from the Science and Technology Bureau of China and the Department of Wildlife Administration is not necessary when the fish in question are neither rare nor endangered (first- or second-class state protection levels). The fish involved in this experiment were approved by the Biomedical Research Ethics Committee of Hunan Normal University (approval number: 2023 No. (610)). The fish were strongly anesthetized with 100 mg/L MS-222 (Sigma-Aldrich, St. Louis, MO, USA) before dissection. Fish caretakers and experimenters were certified under a professional training course for laboratory animal practitioners held by the Institute of Experimental Animals, Hunan Province, China.

### 2.2. Hybrid Lineage Establishment and Sample Collection

**Figure 1:**
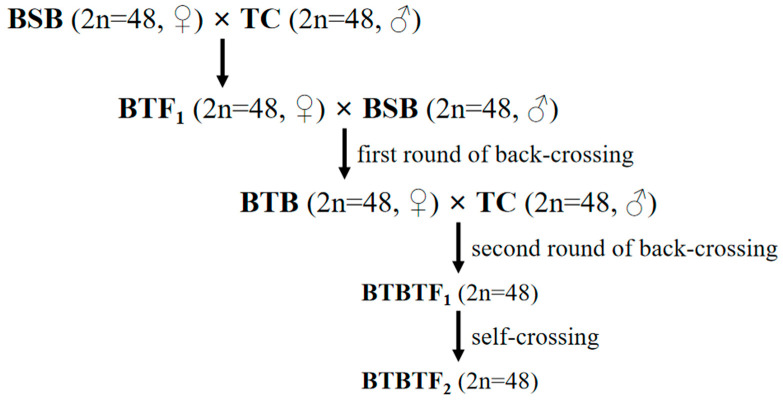
Establishment procedure for the hybrid lineage of BTBTF_1_-F_2_.

The fish specimens used in this study were obtained from the State Key Laboratory of Developmental Biology of Freshwater Fish, Hunan Normal University. During the spawning season (May–July) of 2022, 20 sexually mature female and male BTBTF_1_ individuals were randomly selected. Female fish were injected with luteinizing hormone-releasing hormone analog (LHRH-A) and human chorionic gonadotropin (HCG) at doses of 9.5 μg/kg and 550 IU/kg, respectively. Subsequently, male fish were injected with the same type of oxytocic agent but at half the dosage. Afterwards, artificial insemination and subsequent rearing were conducted in accordance with the method described in [21]. Once they could swim freely, the hatched BTBTF_2_ fries were transferred to a pre-fertilized pool enriched with soybean milk for cultivation. The preparation process of BTBTF_1_-F_2_ is illustrated in Figure 1.

### 2.3. Measurement of DNA Content and Preparation of Chromosomal Metaphase Spreads

To determine the ploidy levels of BTBTF_1_-F_2_, 50 samples each of BSB, TC, BTBTF_1,_ and BTBTF_2_ were selected for DNA content analysis and chromosome preparation. Venous blood (0.2 mL) was collected from the dorsal vein of each fish and preserved in ACD (Acid–Citrate–Dextrose Solution). Following a previously defined protocol [21], blood samples were stained with 4′6-diamidino-2-phenylindole, and the average DNA content of each sample was measured using a flow cytometer (Cell Counter Analyzer, Partec, North Rhine-Westphalia, Germany). This method aligns with the FAO’s *Genomic Characterization of Animal Genetic Resources: Practical Guide* [22]. Compared to the average DNA contents of BSB and TC, the deviation of the ratio of DNA content of BTBTF_1_-F_2_ to the sum of that of BSB and TC from the expected ratio was detected through χ^2^ tests with Yates’ correction. Based on this, BSB, TC, BTBTF_1,_ and BTBTF_2_ individuals, exhibiting no difference in DNA content, were selected for preparing kidney chromosomes following a previously described method [23]. Ten individuals from each fish species were selected for chromosome preparation. Chromosomes in samples that met the following criteria were counted under an optical microscope: clear with distinguishable arm boundaries, clearly visible centromeres, uniform dispersion, no obvious structural aberrations (such as breakage, deletion, and translocation), and the ability to be independently presented., Ten metaphase spreads were observed in each sample, resulting in 100 metaphase spreads recorded for each fish species.

### 2.4. Analysis of Morphological Traits

At the age of 24 months, 30 fish each from BSB, TC, BTBTF_1_, and BTBTF_2_ were selected for morphological trait examination. The full-length-to-body length (FL/BL), body length-to-body height (BL/BH), body length-to-head length (BL/HL), head length-to-head height (HL/HH), body height-to-head height (BH/HH), and caudal peduncle length-to-caudal peduncle height (CPL/CPH) ratios were measured and calculated. For countable traits, the number of scales in lateral, lower lateral, and upper lateral lines, and the number of rays in abdominal, dorsal, and anal fins were counted. The data were averaged and analyzed by analysis of variance and pairwise comparisons using the Statistical Package for the Social Sciences software (version 17.0; SPSS; Chicago, IL, USA).

### 2.5. DNA Extraction, Amplification, and Sequencing of ITS

Genomic DNA from the whole genome was extracted from 20 mg of prepared liver tissue. Liver tissues were obtained from three fish each of BSB, TC, BTBTF_1_, and BTBTF_2_. MiniBEST Universal Genomic DNA Extraction Kit Ver.5.0 (Takara, Beijing, China) was used for the extraction, following the manufacturer’s instructions. DNA quality was evaluated by agarose gel electrophoresis. Once DNA extraction yielded satisfactory results, the distinct differences in ITS region sequences of 45S rDNA between the original parental species BSB and TC were used to identify the specific genetic composition and variations in BTBTF_1_ and BTBTF_2_ generations. Degenerate primers designed by Xiao Jun [24] for the ITS1 region of BSB and TC were employed. The forward and reverse primers were 5′-AGTCGTAACAAGGTTTCCGTAG-3′, and 5′-ATC(A/G)ATGTGTCCTGCAATTCAC-3′, respectively. The total volume of the PCR reaction was 10 μL, consisting of 1 μL of DNA template, 3 μL of ultrapure water, 0.5 μL of reverse primer, 0.5 μL of forward primer, and 5 μL of LA PCR Master Mix (TaKaRa, Beijing, China). The PCR program was as follows: pre-denaturation at 94 °C for 5 min; 35 cycles of 95 °C for 30 s, 52.6 °C for 30 s, and 72 °C for 30 s; termination at 72 °C for 7 min; storage at 4 °C. Subsequently, agarose gel electrophoresis was performed for detection. The correct ITS target fragments of BSB, TC, BTBTF_1_, and F_2_ were recovered using a gel extraction kit (Sangon Biotech, Shanghai, China) for ligation. The ligation mixture employed the pMD18-T vector (TaKaRa, Beijing, China) and contained 2.5 μL of gel-extracted product, 2 μL of solution buffer, and 0.5 μL of pMD18-T vector. The ligation product was sent to Tsingke Biotechnology (Beijing, China) for sequencing, followed by sequence alignment analysis using BioEdit software v7.2.5.

### 2.6. MSAP Analysis

To investigate the inheritance and variation in cytosine methylation between BTBTF_1_-F_2_, BSB, and TC, MSAP analysis was performed as previously described [25]. A total of 500 ng of DNA extracted from each sample was subjected to double-restriction with *EcoR*I/*Msp*I and *EcoR*I/*Hpa*II. Digested DNA was ligated into adaptors (Table 1) using T4 DNA ligase. Subsequently, preselective and selective amplification reactions were performed using primers listed in Table 1. Denaturing polyacrylamide gels (8%) were used to isolate selective amplification products, which were visualized by silver staining. Clear bands were captured for statistical analysis.

*Msp*I and *Hpa*II are a pair of isoschizomers that digest CCGG sites; *Hpa*II is sensitive to cytosine methylation, whereas *Msp*I is not. Consequently, CCGG sites with different methylation statuses produce bands of different sizes after double digestion with *EcoR*I/*Msp*I (abbreviated as M) and *EcoR*I/*Hpa*II (abbreviated as H). Polyacrylamide electrophoresis produced four results corresponding to the methylation pattern. Type 1: nonmethylated (bands in M and H lanes); Type 2: fully methylated (bands in M lane but not in H lane); Type 3: hemimethylated (bands in H lane but not in M lane); Type 4: hypermethylated or lacking CCGG sites (no bands in either lane). Type 4 was excluded from analysis because the absence of CCGG sites may introduce errors in statistical interpretations.

### 2.7. Transcriptome Analysis

The transcriptome of liver tissue from three fish each of BSB, TC, BTBTF_1,_ and BTBTF_2_ was isolated, as previously described [26], and sequenced to analyze homologous gene expression patterns in BTBTF_1_-F_2_. RNA quality was tested using an Agilent 2100 (Agilent Technologies, Santa Clara, CA, USA). Qualified RNA was sequenced using the second-generation sequencing platform (Illumina HiSeq 2500, Illumina Inc., SanDiego, CA, USA). Paired-end de novo was performed to obtain transcripts and unigenes. The CD-HIT software v4.8.1 (threshold = 95%) was used for clustering and de-redundancy to obtain a final set of unigenes. The clean readings of each sample were mapped to unigenes, and FPKM values of unigenes were calculated. The differentially expressed genes (DEGs) of BTBT_1_ and BTBTF_2_ were screened in the homologous gene set using DESeq2, with |Log2 (Fold change) | > 1 and FDR < 0.05. DEG expressions in BTBTF_1_ and BTBTF_2_ were analyzed and compared to those in BSB and TC. The expression patterns of DEGs were classified into five types based on the comparison results: upregulation, in which expression level in BTBTF_1_-F_2_ was higher than in BSB and TC; ELD-BSB, in which expression level in BTBTF_1_-F_2_ was biased towards BSB and was significantly different from TC; ELD-TC, in which expression level in BTBTF_1_-F_2_ was biased towards TC and was significantly different from BSB; downregulation, in which expression level in BTBTF_1_-F_2_ was lower than in BSB and TC; and additivity, in which expression level in BTBTF_1_-F_2_ was intermediate between BSB and TC. DEGs expressed in BTBTF_1_-F_2_ according to the above expression patterns were counted. To verify the homologous gene expression patterns, two representative genes were screened for each expression pattern using real-time quantitative fluorescence PCR (RT-PCR). Primers (Table 2) were designed via Primer Premier 5 software.

## 3. Results

### 3.1. Hybrid Lineage Establishment

In the present study, the BTBTF_1_-F_2_ lineage was successfully prepared by self-mating of bisexual fertile BTBTF_1_ individuals.

### 3.2. DNA Content and Chromosome Number

**Figure 2:**
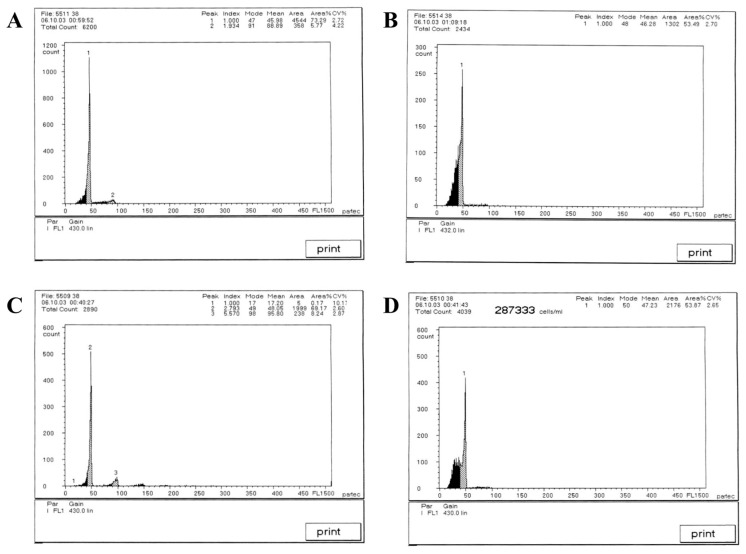
Cytometric histograms of DNA fluorescence for BSB, TC, and BTBTF_1_-F_2_. (**A**) The mean relative fluorescence intensity of BSB (peak 1: 45.98). (**B**) The mean relative fluorescence intensity of TC (peak 1: 46.28). (**C**) The mean relative fluorescence intensity of BTBTF_1_ (peak 2: 48.05). (**D**) The mean relative fluorescence intensity of BTBTF_2_ (peak 1: 47.23).

**Figure 3:**
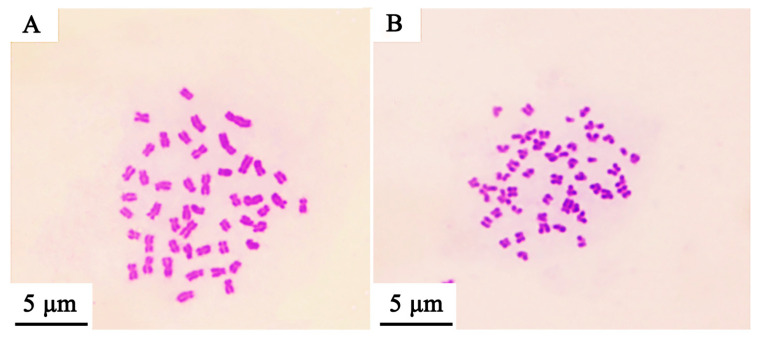

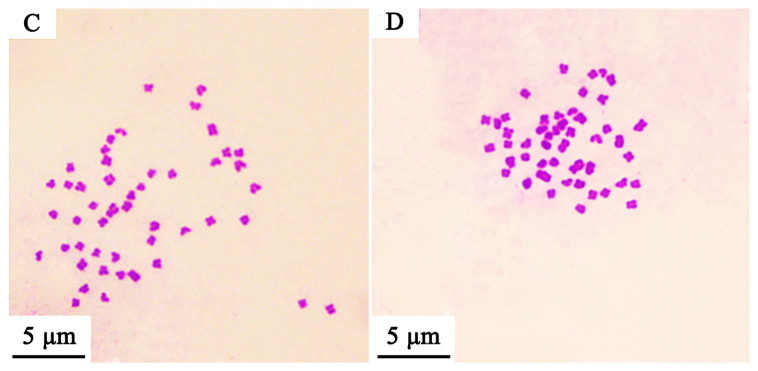
Chromosome spreads during metaphase in BSB, TC, and their hybrid lineage (BTBTF_1_-F_2_). (**A**) Chromosomal spread of BSB. (**B**) Chromosomal spread of TC. (**C**) Chromosomal spread of BTBTF_1_. (**D**) Chromosomal spread of BTBTF_2_. Bar = 5 μm.

Based on establishing the new hybrid *Culter* lineage, the mean DNA content was measured using a flow cytometer, and the results are illustrated in Figure 2 and Table 3. The mean relative fluorescence intensities of BSB, TC, BTBTF_1,_ and BTBTF_2_ were 45.98, 46.28, 48.05, and 47.23, respectively. The ratios of BTBTF_1_/BSB, BTBTF_1_/TC, BTBTF_2_/BSB, BTBTF_2_/TC, and BTBTF_2_/BTBTF_1_ were 1.05, 1.04, 1.03, 1.02, and 0.98, respectively. Comparative analysis revealed no significant differences in the DNA content of BSB, TC, BTBTF_1,_ and BTBTF_2_ (*p* > 0.05), indicating that their DNA contents were comparable. Chromosomal distributions are demonstrated in Figure 3 and Table 4. In total, 100 micrographs of mitotic metaphase chromosomes exhibited that 90% of BSB and 87% of TC had 48 chromosomes, and that the percentages of chromosomes of BTBTF_1_-F_2_ blood cells in mitotic metaphase were 92% and 88%, respectively. Those results showed that BTBTF_1_-F_2_ was examined as a diploid lineage with 48 chromosomes.

### 3.3. Morphological Traits

The specific data for measurable and countable BTBTF_1_ and BTBTF_2_ traits are presented in Table 5 and Table 6, respectively. Except for BL/BH and BH/HH, all measurable traits of BTBTF_2_ were not significantly different from those of BTBTF_1_ (*p* > 0.05). BL/BH, HL/HH, BH/HH, and CPL/CPH ratios of BTBTF_1_ and BTBTF_2_ were between those of BSB and TC. Additionally, BTBTF_1_ and BTBTF_2_ were more inclined towards BSB in some measurable traits, such as BL/HL and BH/HH ratios, and showed a significant difference (*p* < 0.01) compared to TC. All countable traits of BTBTF_1_ and BTBTF_2_ were intermediate between those of BSB and TC, and the numbers of dorsal, abdominal, and anal fin rays of BTBTF_2_ were not significantly different (*p* > 0.05) from those of BTBTF_1_. Additionally, BTBTF_1_ and BTBTF_2_ were more inclined towards BSB in dorsal fin rays, and showed a significant difference (*p* < 0.01) compared to TC.

### 3.4. ITS Sequence Sequencing Results and Analysis

**Figure 4:**
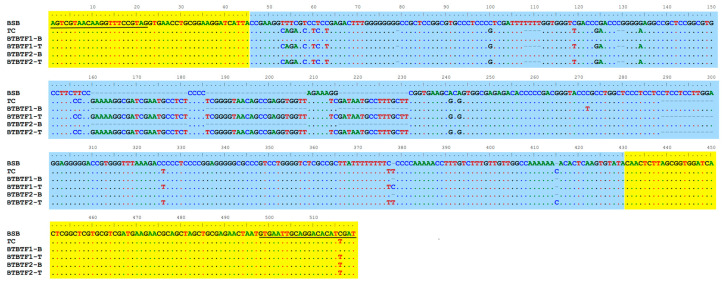
The alignment results of ITS1, partial 18S rDNA, and 5.8S rDNA sequences from BSB, TC, BTBTF_1_, and F_2_ are presented. The black-underlined sequences represent primers. The yellow regions indicate partial 18S and 5.8S rDNA sequences, and the blue region denotes the ITS1 sequence.

ITS1 sequences of BTBTF_1_, BTBTF_2_, and their parents (BSB and TC) were amplified and sequenced using designed primers, with sequences analyzed via BioEdit software v7.2.5. Key results are presented in Figure 4. Partial 18S rDNA (45 bp) and 5.8S rDNA (89 bp) sequences of BTBTF_1_ and F_2_ were consistent with those of BSB and TC. Meanwhile, parental ITS1 sequences differed significantly: BSB exhibited a 322 bp ITS1 sequence, but TC displayed a 366 bp ITS1 sequence. Two parental-derived ITS1 types were identified in both BTBTF_1_ and F_2_—designated as BTBTF_1_-B/T and BTBTF_2_-B/T, respectively—with one exhibiting high similarity (≥99%) to the maternal “BSB-type” ITS1 and the other demonstrating high similarity (≥98.5%) to the paternal “TC-type” ITS1. Additionally, ITS1 sequences of BTBTF_1_ and F_2_ were highly conserved, exhibiting no more than three site-specific differences. This result confirmed that BTBTF_1_ and F_2_ were allodiploids with heterozygous genomes that had inherited genetic material from both parents simultaneously and exhibited stable inheritance.

### 3.5. MSAP Analysis

A total of 7538 bands were obtained after MSAP selective amplification. The results are presented in Table 7. In the BSB genome, 53.70% of CCGG sites were methylated, including 38.21% fully methylated and 15.49% hemimethylated. In the TC genome, 47.88% of CCGG sites were methylated, including 29.53% fully methylated and 18.35% hemimethylated. In BTBTF_1_, 48.01% of CCGG sites were methylated, including 23.43% fully methylated and 24.58% hemimethylated. In BTBTF_2_, 45.40% of CCGG sites were methylated, including 22.06% fully methylated and 23.34% hemimethylated. The genomic methylation levels of BTBTF_1_ and BTBTF_2_ were similar and lower than those of BSB and TC, where the percentage of fully methylated levels was lower than that of the original parents, but the hemimethylation was higher than that of the original parents.

To further study the inheritance and variation in methylation at the CCGG sites of BTBTF_1_ and BTBTF_2_, BTBTF_1_ and BTBTF_2_ bands were compared with those of BSB and TC, respectively. The bands were divided into four classes (A, B, C, and D), and each class was further divided into three subclasses (A1–A3, B1–B3, C1–C3, and D1–D3). The data are presented in Table 8. The bands in class A were the same for BSB and TC, indicating that the methylation sites were inherited. Bands in class B were the same as BSB but not with TC, indicating that the methylation sites were inherited from BSB only. Bands in class C were the same as those in TC but not with BSB, indicating that the methylation sites were inherited from TC only. Bands in class D were inconsistent with BSB and TC, suggesting genomic methylation variation. In BTBTF_1_-F_2_, the percentage of class A was the highest at 37.13% and 38.83%, respectively. In BTBTF_1_-F_2_, the percentage of class C (30.46% and 26.62%) was significantly higher than that of class B (12.94% and 12.31%) (*p* < 0.05), exhibiting bias towards TC. Class D bands were found in BTBTF_1_ and BTBTF_2_, accounting for 19.47% and 22.24%, respectively. These results showed that BTBTF_1_-F_2_ inherited genomes from BSB and TC and produced mutated genes simultaneously.

### 3.6. Transcriptome Analysis

Clean data of 81.36 Gb were obtained by performing next-generation transcriptome sequencing on each of the three fish of BSB, TC, BTBTF_1,_ and BTBTF_2_. The effective data for each sample ranged from 5.85 to 7.06 Gb. The mean GC content was 46.74%. A total of 52,874 unigenes were obtained, with a total length of 62,335,273 bp and an average length of 1178.94 bp.

**Figure 5:**
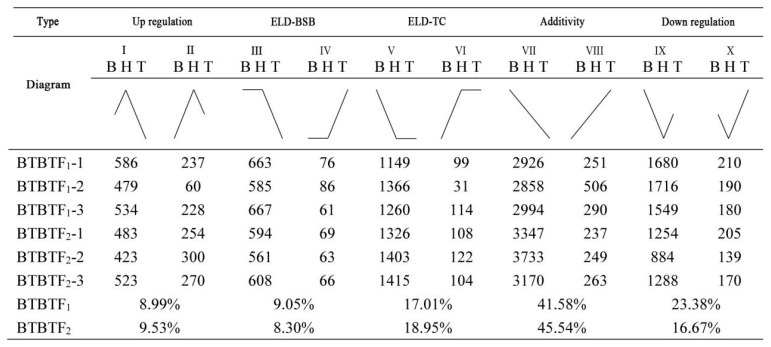
Statistics of expression patterns of homologous genes in BSB, TC, BTBTF_1,_ and BTBTF_2_.

**Figure 6:**
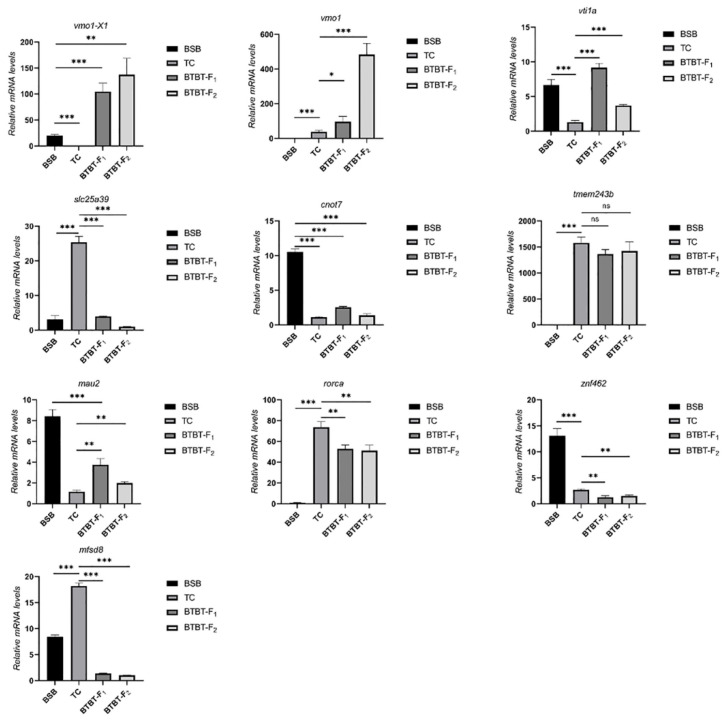
qRT-PCR validation of expression patterns on 10 representative DEGs in the transcriptome of BSB, TC, BTBTF_1,_ and BTBTF_2_. * represents significant difference, ** and *** represents extremely significant difference, and ns represents no significant difference.

Differentially expressed genes (DEGs) were identified using DESeq2, considering the expression levels of each sample. The screening criteria were set as follows: false discovery rate (FDR) < 0.05 and |log_2_ (fold change) | > 1. A total of 7877 DEGs were obtained. The statistics of expression patterns of DEGs in BSB, TC, BTBTF_1,_ and BTBTF_2_ are demonstrated in Figure 5. In BTBTF_1_ and BTBTF_2_, 8.99% and 9.53% of DEGs were upregulated; 9.05% and 8.30% were expressed in ELD-BSB; 17.01% and 18.95% were expressed in ELD-TC; 41.58% and 45.54% were expressed in additivity; and 23.38% and 16.67% were downregulated, respectively. The results exhibited that DEG expression was similar in BTBTF_1_ and BTBTF_2_. Moreover, in BTBTF_1_-F_2,_ the expression pattern of homologous genes in BTBTF_1_ and BTBTF_2_ was mainly based on additive expression and was more biased towards TC than towards BSB (*p* < 0.05). The five expression patterns were verified using qRT-PCR, and the results are illustrated in Figure 6. The verification results were consistent with the transcriptome analysis.

## 4. Discussion

Distant hybridization makes it possible to transfer the genome of one species to another, resulting in phenotypic and genotypic changes in progeny [27], which is widely used to produce offspring with heterosis [28] and form lineages [29]. The feasibility of hybridization makes it a widely used practice in fish genetics and breeding [8]. BTBTF_1_ was obtained by crossing and back-crossing of BSB and TC, displaying a TC-biased appearance. Although BSB and TC belong to different genera, these hybrid offspring were fertile, and BTBTF_2_ was obtained via self-crossing of BTBTF_1_, providing new germplasm resources for breeding of Culter. DNA content analysis, chromosome preparation, and morphological traits revealed that BTBTF_1_-F_2_ was diploid with 48 chromosomes and presented a hybrid appearance compared with the original parents.

The internal transcribed spacer (ITS) region of 45S rDNA functions as a molecular marker for hybrid genetic identification, owing to its moderate evolutionary rate, pronounced interspecific divergence, and exceptional environmental conservation [30]. ITS1 sequencing of BTBT allohybridized progenies (F_1_-F_2_) demonstrated retention of dual parental ITS1 haplotypes: one exhibiting elevated sequence homology (≥99%) to the maternal BSB-type, and the other to the paternal TC-type (≥98.5%). This bimodal inheritance pattern unequivocally substantiates BTBT’s allohybrid genomic architecture—paralleling Schumer et al.’s observations in swordtail hybrids and corroborating analytical precision [31]. Remarkably, ITS1 sequences across F_1_ and F_2_ generations manifested profound sequence conservation, with ≤3 site-specific polymorphisms—well within established parameters of intraspecific genomic variation [32]. Crucially, F_2_ progenies exhibited an absence of parental genomic bias. These findings collectively affirm the generational conservation of ITS genomic architecture in BTBT, establishing its status as a stably inherited allodiploid lineage [21]. Thus, ITS1 profiling not only validates BTBT’s allodiploid constitution but also provides a new insight underlying genetic stabilization in distant hybridization, thereby furnishing molecular substantiation for elite strain aquacultural propagation.

DNA methylation plays a critical role in regulating gene expression, developing X-chromosome inactivation, genetic imprinting, and the repression of transposable elements [33]. Previous studies have indicated that it may be associated with gene expression and phenotypic variation in fish [25]. In this study, MSAP results exhibited that BTBTF_1_-F_2_ inherited methylation sites from BSB and TC, consistent with the findings of ITS analysis, further indicating that BTBTF_1_-F_2_ is a hybrid lineage with a hybrid genome. Conversely, the genomic methylation level of BTBTF_1_-F_2_ was lower than that of its original parents, consistent with a related study [34] and international reports on hybrid fish epigenetics [18]. Further research is needed to determine whether BTBTF_1_-F_2_ exhibits more hybrid vigor than BSB and TC, or if this vigor correlates with reduced genomic methylation levels.

Cytoplasmic inheritance frequently engenders a pronounced bias in genetic expression within hybrid offspring, preferentially favoring the maternal lineage [35]. For example, a marked preference for maternal transcripts was observed in the placenta of mid-gestation F_1_ hybrid mice derived from a cross between *Mus musculus* (♀) × *Rattus norvegicus* (♂) [36]. However, DEG expression in BTBTF_1_-F_2_ was biased towards that of the original male parent (TC) in this study. These findings demonstrated that bias in homologous gene expression within hybrid progeny does not stem exclusively from cytoplasmic inheritance, but may be significantly modulated by genomic elements, particularly paternal DNA, thereby inducing a preference for paternal expression.

## 5. Conclusions

In sum, we established a hybrid culter lineage from *Megalobrama amblycephala* (♀) and *Culter alburnus* (♂), evacuated the biological characteristics regarding the DNA content, chromosome number, morphological traits, ITS1, MSAP, and transcriptomic analyses. And the results of BTBTF_2_ were consistent with those of BTBTF_1_, indicating that BTBTF_1_-F_2_ exhibited stable genetic traits. The successful preparation of BTBTF_1_-F_2_ demonstrated the fertility of this type of hybrid fish, providing a novel fish resource for genetic and breeding research in *Culter*.

## Figures and Tables

**Table 1:** Adaptor and primer sequences used in MSAP.

Adaptor/Primer Name	Sequence (5′-3′)
*EcoR*I-adaptor 1	CTCGTAGACTGCGTACC
*EcoR*I-adaptor 2	AATTGGTACGCAGTCTAC
*Msp*I/*Hpa*II-adaptor 1	GACGATGAGTCTAGAA
*Msp*I/*Hpa*II-adaptor 2	CGTTCTAGACTCATC
*EcoR*I- preselective primer	GACTGCGTACCAATTCA
*Msp*I/*Hpa*II- preselective primer	GATGAGTCTAGAACGGT
*EcoR*I-selective primer 1	GACTGCGTACCAATTCAAT
*EcoR*I-selective primer 2	GACTGCGTACCAATTCAGA
*EcoR*I-selective primer 3	GACTGCGTACCAATTCAGC
*EcoR*I-selective primer 4	GACTGCGTACCAATTCAGT
*EcoR*I-selective primer 5	GACTGCGTACCAATTCACA
*Msp*I/*Hpa*II-selective primer 1	GATGAGTCTAGAACGGTAG
*Msp*I/*Hpa*II-selective primer 2	GATGAGTCTAGAACGGTAC
*Msp*I/*Hpa*II-selective primer 3	GATGAGTCTAGAACGGTAT
*Msp*I/*Hpa*II-selective primer 4	GATGAGTCTAGAACGGTGA
*MSP*I/*HPA*II-selective primer 5	GATGAGTCTAGAACGGTGC
*MSP*I/*HPA*II-selective primer 6	GATGAGTCTAGAACGGTGT
*MSP*I/*HPA*II-selective primer 7	GATGAGTCTAGAACGGTCA
*MSP*I/*HPA*II-selective primer 8	GATGAGTCTAGAACGGTCG

**Table 2:** Real-time fluorescence quantitative PCR primers.

Pattern Gene Primers	Primer Sequence (5′-3′)
*vmo1-X1*-F	GTGACGAATGGAATGGAGCAG
*vmo1-X1*-R	CCACCTTCAAACGAAACCCT
*vmo1*-F	ATGCGTCGCCTCCTTTCC
*vmo1*-R	TGTGCTCCACTCATCTGGTCTTAC
*vti1a*-F	CCTGGAGGTTCGTGAGATTC
*vti1a*-R	CTGTCCCACTTGCTCGGTTT
*slc25a39*-F	TTTCAGCCCTTTACTGGTTT
*slc25a39*-R	CTGGATTTGCCTGCGTGTT
*cnot7*-F	CCTCAGCATCAGGCAGGGTC
*cnot7*-R	CGCAATACTTGGCATCATCG
*tmem243b*-F	TCTTCCCTTCACTGCCTCCC
*tmem243b*-R	TCGCCCTGCCTATACCAAAA
*mau2*-F	TTGCTGTTGATGGAGCGTAAG
*mau2*-R	TGGGAAGCCAGTGGAAGAGG
*rorca*-F	CTGGATGACATAACCACGCTACC
*rorca*-R	GGAGAACTTTGGGAGGACGAA
*znf462*-F	ACAACGGGATTGAAGGGTCAG
*znf462*-R	TACGGACGAGGTCGGCATT
*mfsd8*-F	GAGTCCATCCTCGTCTTCCTG
*mfsd8*-R	GAATGTCTGCCCACTGTATTGTAG

**Table 3:** Mean DNA contents of BSB, TC and BTBTF_1_-F_2_.

Fish Type	Mean DNA Content	Observed	Expected
BSB	45.98		
TC	46.28		
BTBTF_1_	48.05	BTBTF_1_/BSB = 1.05 ^a^	1
		BTBTF_1_/TC = 1.04 ^a^	1
BTBTF_2_	47.23	BTBTF_2_/BSB = 1.03 ^a^	1
		BTBTF_2_/TC = 1.02 ^a^	1
		BTBTF_2_/BTBTF_1_ = 0.98 ^a^	1

^a^: no significant difference between the observed and expected values (*p* > 0.05).

**Table 4:** Examination of chromosome number of BSB, TC and BTBTF_1_-F_2_.

Fish Type	Distribution of Chromosome Number			Proportion of Phases with 48 Chromosomes
	<48	48	>48	
BSB	6	90	4	90%
TC	8	87	5	87%
BTBTF_1_	6	92	2	92%
BTBTF_2_	11	88	1	88%

**Table 5:** The measurable traits of BSB, TC and BTBTF_1_-F_2_.

	Fish Type	FL/BL	BL/BH	BL/HL	HL/HH	BH/HH	CPL/CPH
Mean ± Standard Deviation	BSB	1.17 ± 0.03	2.36 ± 0.04	4.52 ± 0.21	1.21 ± 0.12	2.10 ± 0.21	1.07 ± 0.10
	TC	1.16 ± 0.02	4.02 ± 0.23	5.11 ± 0.25	2.05 ± 0.25	2.37 ± 0.12	1.24 ± 0.12
	BTBTF_1_	1.18 ± 0.03	3.80 ± 0.15	4.48 ± 0.19	1.80 ± 0.11	2.11 ± 0.06	1.13 ± 0.05
	BTBTF_2_	1.18 ± 0.02	3.71 ± 0.11	4.58 ± 0.21	1.81 ± 0.24	2.16 ± 0.08	1.15 ± 0.07
*p* value	BSB vs. TC	>0.05	<0.01	<0.01	<0.01	<0.01	<0.01
	BSB vs. BTBTF_1_	>0.05	<0.01	>0.05	<0.01	>0.05	0.02
	BSB vs. BTBTF_2_	0.03	<0.01	>0.05	<0.01	>0.05	<0.01
	TC vs. BTBTF_1_	>0.05	<0.01	<0.01	<0.01	<0.01	<0.01
	TC vs. BTBTF_2_	<0.01	<0.01	<0.01	<0.01	<0.01	<0.01
	BTBTF_1_ vs. BTBTF_2_	>0.05	0.02	>0.05	>0.05	0.02	>0.05

**Table 6:** The countable traits of BSB, TC and BTBTF_1_-F_2_.

	Fish Type	Lateral Scales	Upper Lateral Scales	Lower Lateral Scales	Dorsal Fin rays	Abdominal Fin Rays	Anal Fin Rays
Range of countable traits	BSB	49–53	9–11	9–12	III + 8–9	8–10	III + 24–27
	TC	82–91	16–18	6–7	III + 7	9	III + 21–23
	BTBTF_1_	66–71	12–15	9–11	III + 8–9	9	III + 23–26
	BTBTF_2_	64–68	14–16	10–11	III + 8–9	9	III + 23–25
*p* value	BSB vs. TC	<0.01	<0.01	<0.01	<0.01	>0.05	<0.01
	BSB vs. BTBTF_1_	<0.01	<0.01	<0.01	>0.05	>0.05	>0.05
	BSB vs. BTBTF_2_	<0.01	<0.01	>0.05	>0.05	>0.05	<0.01
	TC vs. BTBTF_1_	<0.01	<0.01	<0.01	<0.01	>0.05	<0.01
	TC vs. BTBTF_2_	<0.01	<0.01	<0.01	<0.01	>0.05	<0.01
	BTBTF_1_ vs. BTBTF_2_	<0.01	<0.01	<0.01	>0.05	>0.05	>0.05

**Table 7:** Methylation degree of BSB, TC, BTBTF_1_-F_2_.

Fish Type	Type 1	Type 2	Type 3	Total	Percent of Type 1 (%)	Percent of Type 2 (%)	Percent of Type 3 (%)
BSB	532	439	178	1149	46.30	38.21	15.49
TC	676	383	238	1297	52.12	29.53	18.35
BTBTF_1_	1358	612	642	2612	51.99	23.43	24.58
BTBTF_2_	1354	547	579	2480	54.60	22.06	23.34

**Table 8:** Genetic and variation status of methylation in BTBTF_1_ and BTBTF_2_.

Fish Type	Class	Subclass	Total Number in Class	Total Number in Subclass	Percent of Class (%)	Percent of Subclass (%)
BTBTF_1_	A	A1	740	287	37.13	14.40
		A2		141		7.07
		A3		312		15.66
	B	B1	258	113	12.94	5.67
		B2		48		2.40
		B3		97		4.87
	C	C1	607	297	30.46	14.92
		C2		156		7.82
		C3		154		7.72
	D	D1	388	121	19.47	6.07
		D2		151		7.58
		D3		116		5.82
BTBTF_2_	A	A1	700	272	38.83	15.09
		A2		120		6.66
		A3		308		17.08
	B	B1	222	102	12.31	5.66
		B2		39		2.16
		B3		81		4.49
	C	C1	480	196	26.62	10.87
		C2		149		8.26
		C3		135		7.49
	D	D1	401	129	22.24	7.15
		D2		119		6.60
		D3		153		8.49

## Data Availability

The complete clean reads for the libraries used in this study have been uploaded to the NCBI Sequence Read Archive (SRA) site (http://www.ncbi.nlm.nih.gov/sra/ (accessed on 25 September 2025); BioProject ID PRJNA1353644).

## References

[1] Zhu D., Li Q., Wang G., Sun Y., Chen J., Li P. (2016). The complete mitochondrial genome of the hybrid of Culter alburnus (♀) × Ancherythroculter nigrocauda (♂). Mitochondrial DNA.

[2] Zhang G., Fang D., Xue X., Zhang M., Feng X., Yang X. (2021). *Culter alburnus* Basilewsky in different populations: Genetic diversity analysis based on Cytochrome B (Cyt b) gene. Chin. Agric. Sci. Bull..

[3] Huang Y., Gong W., Ren H., Xiong J., Gao X., Sun X. (2017). Identification of the conserved and novel microRNAs by deep sequencing and prediction of their targets in *Topmouth culter*. Gene.

[4] Wang Y., Ren L., Xu D.-P., Fang D.-A. (2022). Exploring the trophic niche characteristics of four carnivorous *Cultrinae fish* species in Lihu Lake, Taihu Basin, China. Front. Ecol. Evol..

[5] Chen J., Luo M., Li S., Tao M., Ye X., Duan W., Zhang C., Qin Q., Xiao J., Liu S. (2018). A comparative study of distant hybridization in plants and animals. Sci. China Life Sci..

[6] Rahman M.A., Lee S.G., Yusoff F.M., Rafiquzzaman S. (2018). Hybridization and its application in aquaculture. Sex Control Aquac..

[7] Wang M., Ou Y., Guo Z., Li J., Li H., Li X., Li J., Wang S., Liu Q., Wang J. (2024). Characterization of allodiploid and allotriploid fish derived from hybridization between *Cyprinus carpio haematopterus* (♀) and *Gobiocypris rarus* (♂). Reprod. Breed..

[8] Zhang X., Liu F., Li B., Yu J., Duan L., Huang Z., Zhou Z., Shu Y., Lin J., Xiong X. (2024). Comparative analysis of nutrients in muscle and ovary between an improved fish and its parents. Reprod. Breed..

[9] Goswami M., Kuchay M.A. (2023). Chapter-12 Hybridization: Importance, Techniques and Consequences. Recent Trends Agric..

[10] Qin Q., Wang Y., Wang J., Dai J., Xiao J., Hu F., Luo K., Tao M., Zhang C., Liu Y. (2014). The autotetraploid fish derived from hybridization of *Carassius auratus* red var. (female)× *Megalobrama amblycephala* (male). Biol. Reprod..

[11] Chen S., Wang J., Liu S., Qin Q., Xiao J., Duan W., Luo K., Liu J., Liu Y. (2009). Biological characteristics of an improved triploid crucian carp. Sci. China Ser. C Life Sci..

[12] Gan Y.M. (2011). Identification of Individual Chromosomes and Localization of rDNA in Tetraploid Cotton Species and Their Donor Genomes. PhD. Thesis.

[13] Péter P., Jaakko H. (2010). Nuclear ribosomal spacer regions in plant phylogenetics: Problems and prospects. Mol. Biol. Rep..

[14] Gaut B., Tredway L., Kubik C., Gaut R., Meyer W. (2000). Phylogenetic relationships and genetic diversity among members of the Festuca-Lolium complex (Poaceae) based on ITS sequence data. Plant Syst. Evol..

[15] Wyatt P., Pitts C., Butlin R. (2006). A molecular approach to detect hybridization between bream Abramis brama, roach Rutlius rutilus and rudd Scardinius erythrophthalmus. J. Fish Biol..

[16] Park S.-Y., Murthy H., Chakrabarthy D., Paek K.-Y. (2009). Detection of epigenetic variation in tissue-culture-derived plants of Doritaenopsis by methylation-sensitive amplification polymorphism (MSAP) analysis. Vitr. Cell. Dev. Biol.-Plant.

[17] Peraza-Echeverria S., Herrera-Valencia V.A., Kay A.-J. (2001). Detection of DNA methylation changes in micropropagated banana plants using methylation-sensitive amplification polymorphism (MSAP). Plant Sci..

[18] Shao T., Yuan Z., Qing X., Dong S., Ying Y. (2007). Comparison of methylation level of genomes among different animal species and various tissues. Chin. J. Agric. Biotechnol..

[19] Zhao Y., Zhou J., Dong Y., Xu D., Qi D. (2024). Transcriptome Analysis Reveals the Molecular Mechanisms Underlying Growth Superiority in a Novel Gymnocypris Hybrid, *Gymnocypris przewalskii*♀× *Gymnocypris eckloni*♂. Genes.

[20] Wu C., Chen Q., Huang X., Hu F., Zhu S., Luo L., Gong D., Gong K., Zhao R., Zhang C. (2019). Genomic and epigenetic alterations in diploid gynogenetic hybrid fish. Aquaculture.

[21] Wu C., Huang X., Chen Q., Hu F., Zhou L., Gong K., Fu W., Gong D., Zhao R., Zhang C. (2020). The formation of a new type of hybrid culter derived from a hybrid lineage of *Megalobrama amblycephala* (♀) × *Culter alburnus* (♂). Aquaculture.

[22] Ajmone-Marsan P., Boettcher P.J., Ginja C., Kantanen J., Lenstra J.A. (2023). Genomic Characterization of Animal Genetic Resources. Practical Guide; Food and Agriculture.

[23] Liu Q., Liu J., Liang Q., Qi Y., Tao M., Zhang C., Qin Q., Zhao R., Chen B., Liu S. (2019). A hybrid lineage derived from hybridization of *Carassius cuvieri* and *Carassius auratus* red var. and a new type of improved fish obtained by back-crossing. Aquaculture.

[24] Xiao J., Fangzhou H., Kaikun L., Li W., Liu S. (2016). Unique nucleolar dominance patterns in distant hybrid lineage derived from *Megalobrama amblycephala* × *Culter alburnus*. BMC Genet..

[25] Xiao J., Song C., Liu S., Tao M., Hu J., Wang J., Liu W., Zeng M., Liu Y. (2013). DNA methylation analysis of allotetraploid hybrids of red crucian carp (*Carassius auratus* red var.) and common carp (*Cyprinus carpio* L.). PLoS ONE.

[26] Liu Q., Qi Y., Liang Q., Xu X., Hu F., Wang J., Xiao J., Wang S., Li W., Tao M. (2018). The chimeric genes in the hybrid lineage of *Carassius auratus cuvieri* (♀) × *Carassius auratus* red var. (♂). Sci. China Life Sci..

[27] Liu Q., Liu J., Yuan L., Li L., Tao M., Zhang C., Qin Q., Chen B., Ma M., Tang C. (2020). The establishment of the fertile fish lineages derived from distant hybridization by overcoming the reproductive barriers. Reproduction.

[28] Hu L., Huang X., Mao J., Wang C., Bao Z. (2013). Genomic characterization of interspecific hybrids between the scallops *Argopecten purpuratus* and *A. irradians irradians*. PLoS ONE.

[29] Zhang Z.H., Chen J., Li L., Tao M., Zhang C., Qin Q., Xiao J., Liu Y., Liu S. (2014). Research advances in animal distant hybridization. Sci. China Life Sci..

[30] Soltis P.S., Soltis D.E. (2000). The role of genetic and genomic attributes in the success of polyploids. Proc. Natl. Acad. Sci. USA.

[31] Schumer M., Cui R., Powell D.L., Rosenthal G.G., Andolfatto P. (2016). Ancient hybridization and genomic stabilization in a swordtail fish. Science.

[32] Allendorf F.W., Leary R.F., Spruell P., Wenburg J.K. (2001). The problems with hybrids: Setting conservation guidelines. Trends Ecol. Evol..

[33] Bommarito P.A., Fry R.C. (2019). The role of DNA methylation in gene regulation. Toxicoepigenetics.

[34] Ou M., Mao H., Luo Q., Zhao J., Liu H., Zhu X., Chen K., Xu H. (2019). The DNA methylation level is associated with the superior growth of the hybrid fry in snakehead fish (*Channa argus*× *Channa maculata*). Gene.

[35] Li W., Liu J., Tan H., Luo L., Cui J., Hu J., Wang S., Liu Q., Hu F., Tang C. (2018). Asymmetric expression patterns reveal a strong maternal effect and dosage compensation in polyploid hybrid fish. BMC Genom..

[36] Finn E.H., Smith C.L., Rodriguez J., Sidow A., Baker J.C. (2014). Maternal bias and escape from X chromosome imprinting in the midgestation mouse placenta. Dev. Biol..

